# Influence of foot orthoses on bone alignment parameters of the foot: a cadaveric weight-bearing CT study

**DOI:** 10.1007/s00402-025-06124-z

**Published:** 2025-12-09

**Authors:** Lara Krüger, Alexander Simon, Eva Goedecke, Carsten Schlickewei, Tanja Spethmann, Leon-Gordian Leonhardt, Michael Hahn, Frank Timo Beil, Tim Rolvien

**Affiliations:** 1 Department of Trauma and Orthopaedic Surgery, University Medical Center Hamburg-Eppendorf, Hamburg, Germany; 2 Institute of Osteology and Biomechanics, University Medical Center Hamburg-Eppendorf, Hamburg, Germany; 3 Institute of Anatomy and Experimental Morphology, University Medical Center Hamburg-Eppendorf, Hamburg, Germany; 4 Helios ENDO-Klinik Hamburg, Hamburg, Germany

**Keywords:** Biomechanics, Flatfoot, Foot orthoses, Insoles, Weight-bearing computed tomography

## Abstract

**Introduction:**

Given the inconclusive evidence regarding the effects of foot orthoses in flatfoot management, this study aimed to assess the impact of two distinct types of foot orthoses on three-dimensional bone alignment of the foot using advanced imaging techniques.

**Materials and methods:**

Sixteen fresh-frozen cadaveric feet showing radiological signs of progressive collapsing foot deformity were collected. Two different types of foot orthoses, type I with medial support and lateral counter-support and type II with an additional metatarsal pad, were custom-made. The feet were loaded using a testing machine and scanned by weight-bearing computed tomography. Semi-automatic 3D models of bone dimensions and axes were generated to compute the foot alignment. This study was designed according to the QUACS scale.

**Results:**

The sagittal talus-first metatarsal angle was corrected by an average of 2.0±3.0 degrees with type I orthoses (*p* = 0.019), and 2.6±3.7 degrees with type II orthoses (*p* = 0.018). The axial talocalcaneal angle was corrected by an average 1.8±2.8 degrees with type I orthoses (*p* = 0.021), and 2.0±3.1 degrees with type II orthoses (*p* = 0.02). The axial talus-first metatarsal angle was corrected by an average 1.3±2.0 degrees with type I orthoses (*p* = 0.024), while type II orthoses caused no significant effect (*p* = 0.297). No significant differences were found between type I and type II orthoses (all *p* > 0.05).

**Conclusions:**

In this cadaveric study, foot orthoses with medial support, with or without a metatarsal pad, improved all angles tested, but the effect appeared to be small. It remains to be determined whether these subtle changes are responsible for the positive clinical effects described in other studies, or whether orthoses have a positive effect even without reaching the intended alignment correction.

## Introduction

The use of foot orthoses is one of the most commonly applied interventions in the field of conservative orthopaedic foot therapy [1]. As a result, considerable socio-economic and patient-specific costs arise. In Germany alone, orthotic and insole treatment resulted in costs of 529 million euros for state health insurance funds in 2022 [2], not including private additional payments or the costs covered by private health insurance funds. Progressive collapsing foot deformity (PCFD), commonly also referred to as flatfoot deformity, is one of the most prevalent pathologies of the human foot and represents one of the main indications for orthotic treatments [1, 3–5]. PCFD is characterized by various deformities, which include collapse of the longitudinal arch, hindfoot valgus, and forefoot abduction [5]. To improve the deformities described, foot orthoses are commonly constructed with a medial longitudinal arch support with lateral counter-support [6]. The integration of a metatarsal pad is optional and intends to provide transverse arch support [7]. However, the available scientific literature regarding foot orthoses for PCFD in adults is inconclusive. While some studies described a positive clinical effect [8–10], a recent systematic literature review found a general lack of evidence [11]. A meta-analysis from the same year concluded that foot orthoses can reduce pain, but not realign foot posture [12]. Controversy also exists on whether using an additional metatarsal pad can improve the effectiveness of foot orthoses. While some studies have described a reduction in plantar forefoot pressure [13–15], others have not observed any positive effect of the metatarsal pad [16, 17]. The effects of a metatarsal pad on bone alignment in PCFD have not yet been investigated.

Weight-bearing cone-beam computed tomography (WBCT) has become increasingly important in recent years. This relatively new technique enables more precise visualization of the bone alignment with superior diagnostic accuracy than conventional radiography [18–20]. In particular, for the assessment of complex multidimensional deformities such as PCFD, WBCT offers an optimal diagnostic tool. Given the considerable socio-economic and individual costs and the critical gap in the literature, further evaluation of the three-dimensional effect of orthoses, moving beyond subjective clinical outcomes, seems urgently needed. The following questions arise: (1) Do foot orthoses have the potential to affect typical bone alignment parameters of PCFD? (2) Can foot orthoses with or without a metatarsal pad influence the bone alignment to a different extent? We hypothesize that the orthoses correct bone alignment and that the metatarsal pad provides an additional superior effect. The findings obtained may help to gain deeper understanding of the effect and corrective potential of orthoses in PCFD.

## Materials and methods

### Study design and specimens

A cadaveric study was designed to avoid exposing live human subjects to increased ionizing radiation when repeatedly analyzing the specimen using WBCT. The study was designed according to the QUACS scale [21]. The tests were supervised and evaluated by two independent researchers, one of whom is a trained and certified foot surgeon and the other one in orthopaedic training. Sixteen fresh frozen lower legs and feet of eight body donors were collected. Only feet expressing PCFD under load after thawing, with a resulting talo-metatarsal-I-angle of more than − 4 degrees, were included. Donors with rigid foot mechanics, a body weight of more than 110 kg or with scars indicating previous surgery were excluded. Four (50%) female and four (50%) male body donors were included. The mean age was 88 (range, 80–94) years. The mean BMI was 24 (range, 15–35) kg/m^2^.

**Fig. 1:**
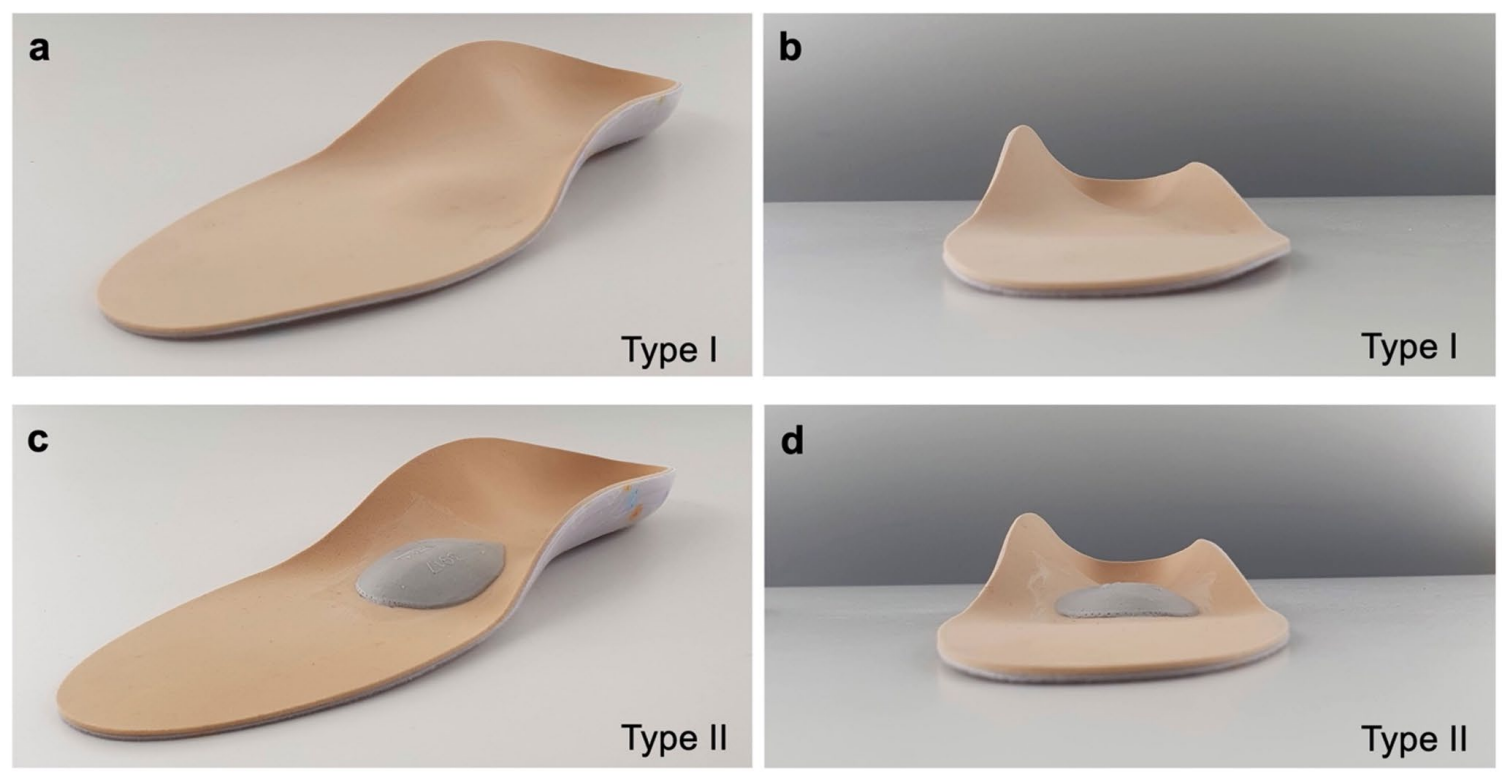
Design of the foot orthoses used. Type I, with medial arch support and lateral countersupport. Type II, with the same medial and lateral support and an additional metatarsal pad. **a** Type I orthosis, oblique frontal view. **b** Type I orthosis, frontal view. **c** Type II orthosis, oblique frontal view. **d** Type II orthosis, frontal view

An unloaded foam box impression of the cadaveric feet was used to create a negative imprint. Based on this, the foot orthoses were custom-made from a polyurethane body, 35 Shore A, with polyethene/polypropylene reinforcement core by a certified orthopaedic technician. The height of the medial support was individually adjusted to the respective foot and ranged between 20 and 30 mm. Two different types of foot orthoses were fabricated for each foot: Type I with medial arch support and lateral counter-support (Fig. 1a and b) and type II with the same medial and lateral support plus an additional metatarsal pad for retrocapital support with a height of 6 mm (Fig. 1c and d).

### Experimental setup

**Fig. 2:**
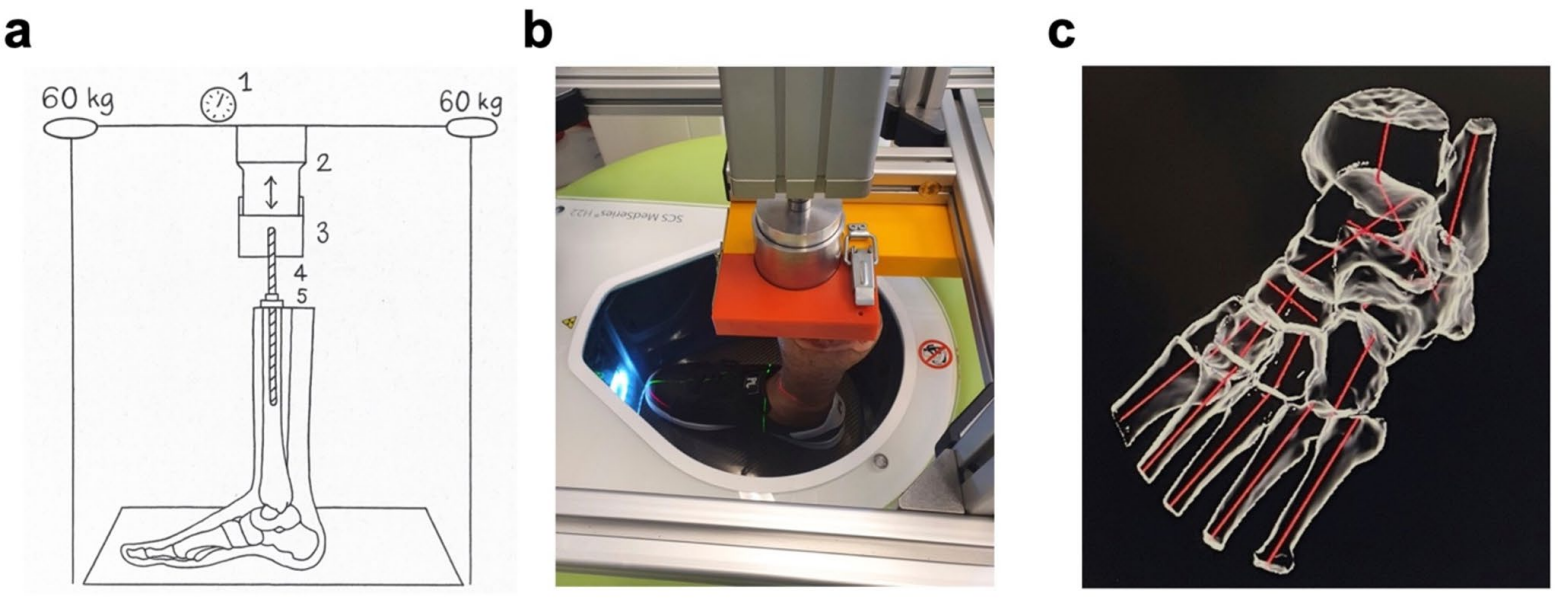
Experimental setup. **a** Schematic illustration of the testing machine. (1) Pressure gauge to measure applied force, (2) piston (3) cranially milled cylinder (4) threaded rod (5) washer and nut. **b** Cadaver foot clamped into the testing machine while wearing a shoe and standing in the WBCT, photographed from above. The silver piston and cylinder can be seen in an additional orange/yellow frame that protects the CT in case the mechanism falls to the side. **c** Exemplary 3D reconstruction of the foot including calculated axes

The feet were mounted into a testing machine, which allowed controlled loading while performing WBCT (SCS MedSeries^®^ H22, SCS Sophisticated Computertomographic Solutions GmbH, Aschaffenburg, Germany) (Fig. 2a and b). A pneumatic piston applied a defined force downwards, simulating the individual body weight of the specimen. A threaded rod was screwed into the tibia and was fixed cranially with a washer and nut to prevent the rod from sintering into the bone. The free end of the rod was screwed into an aluminium cylinder that had a raised rim cranially to achieve a positive fit and guide the pneumatic piston during power transmission. This allowed the piston to be guided in the cylinder, which at the same time allowed minimal clearance for the foot to adapt to the ground under load. The excellent reliability and the adequate load distribution of the test machine have been demonstrated previously [22].

### WBCT imaging protocol

As established before [23], pre-conditioning before testing was performed by loading the feet with half the body weight three times for 10 s. Then unloaded and loaded WBCT images with the feet wearing a soft sneaker shoe (item number 1409823, GSG Great Sports GmbH, Unna, Germany) were taken. Loading was performed with the individual body weight of the body donors. Subsequently, WBCT images were taken under load with orthosis type I and type II inserted in the shoe. The shoe was used to ensure adequate positioning of the foot on the insole.

### WBCT image evaluation and assessment of PCFD

**Fig. 3:**
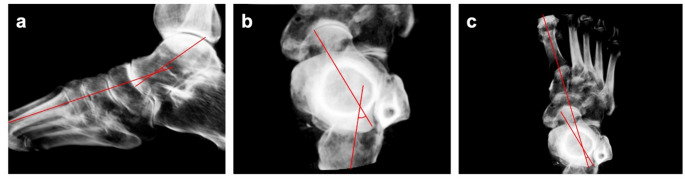
WBCT images showing the semi-automatic determination of three PCFD-related angles. **a** Evaluation of the medial longitudinal arch by determining the sagittal Meary’s angle (SMA), the sagittal talus-first metatarsal angle. **b** Evaluation of the hindfoot alignment by determining the Kite angle, the axial talocalcaneal angle (ATCA). **c** Evaluation of the forefoot abduction by determining the axial Meary’s angle (AMA), defined as the axial talus-first metatarsal angle

The software DISIOR Bonelogic Ortho (release 2.1, IFU version 6.0; Helsinki, Finland) was used for semi-automatic evaluation of the scans. This software has proven to be highly precise and reliable in assessing the dimensions and axes of WBCT scans [24–26]. The software defines anatomical reference frames to calculated bone axes and angles based on the longitudinal axis of the bones, in anatomical plane projections and in 3D (Fig. 2c) [27]. To evaluate PCFD, three angles were selected, that are well-established in assessing the configuration of the medial longitudinal arch, hindfoot alignment, and forefoot abduction [5, 28, 29]. For configuration of the medial longitudinal arch, the sagittal Meary’s angle (SMA), the sagittal talus-first metatarsal angle, was used (Fig. 3a). Hindfoot alignment was determined by the Kite angle, the axial talocalcaneal angle (ATCA) (Fig. 3b). Forefoot abduction was measured by the axial Meary’s angle (AMA), defined as the axial talus-first metatarsal angle **(**Fig. 3c).

### Statistical analysis

Statistical analysis was performed using SPSS Statistics 29.0.2 (IBM, Armonk, NY, USA) and GraphPad Prism 10.2.3 (GraphPad Software, San Diego, CA, USA). Results are reported as mean values with standard deviation. To evaluate normal distribution of the data, the Shapiro-Wilk test was used. A paired two-tailed *t* test for normally distributed data and Wilcoxon signed-rank test for non-normally distributed data was used to compare different groups. Effect sizes for comparisons between two groups were reported as Cohen’s d (0.2 ≙ small, 0.5 ≙ medium, 0.8 ≙ large effect size). The level of statistical significance was defined as *p* < 0.05. The selected sample size aligns with common sample sizes used in biomechanical studies involving cadavers [19, 22, 30–33].

### Ethics

This study was approved by the local ethics committee (2023–300307-WF). All procedures were in accordance with the ethical standards of the 1964 Declaration of Helsinki and its later amendments.

## Results

### Collapse of the longitudinal arch

**Fig. 4:**
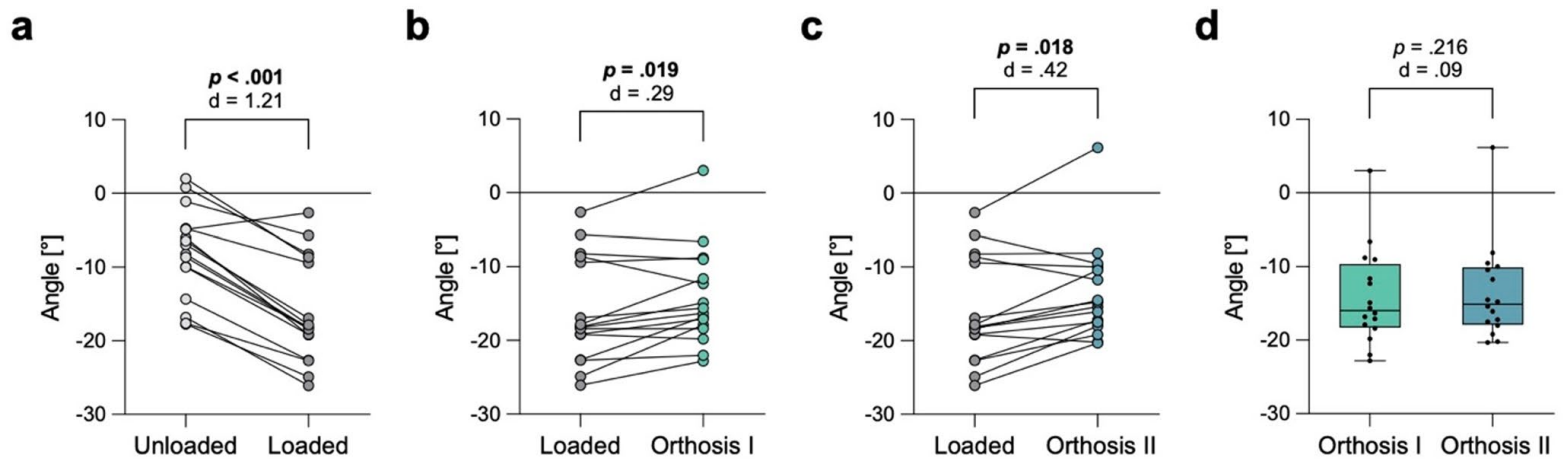
Assessment of collapse of the longitudinal arch by measuring the sagittal Meary’s angle (SMA). **a** Significant decrease of the SMA when loading the feet with full body weight. **b** Significant increase of the SMA when using orthosis type (I) **c** Significant increase of the SMA when using orthosis type (II) **d** No significant difference between orthoses type I and II in the SMA. Exact p-values with corresponding Cohen’s d effect size are displayed

Loading the feet without orthoses resulted in a significant decrease of the SMA by 8.0 ± 3.6 degrees (Fig. 4a). The use of type I orthosis resulted in a significant increase of 2.0 ± 3.0 degrees (Fig. 4b**)**, while type II orthosis resulted in a significant increase of 2.6 ± 3.7 degrees (Fig. 4c). There was no significant difference in the SMA between type I and type II orthoses (Fig. 4d**)**.

### Hindfoot alignment

**Fig. 5:**
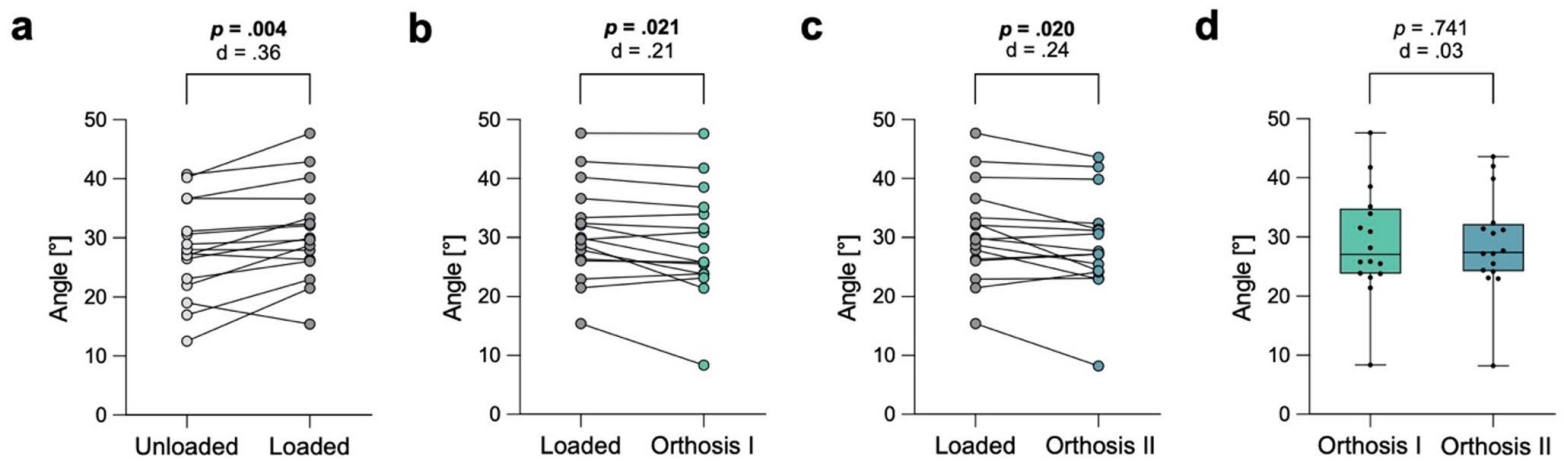
Assessment of hindfoot alignment by measuring the axial talocalcaneal angle (ATCA). **a** Significant increase of the ATCA when loading the feet with full body weight. **b** Significant decrease of the ATCA when using orthosis type (I) **c** Significant decrease of the ATCA when using orthosis type (II) **d** No significant difference between orthoses type I and II in the ATCA. Exact p-values with corresponding Cohen’s d effect size are displayed

Loading the feet without orthoses led to an increase of the ATCA by a mean of 2.9 ± 3.5 degrees **(**Fig. 5a**)**. The use of the type I orthosis led to a significant decrease of 1.8 ± 2.8 degrees **(**Fig. 5b**)**, while type II orthosis led to a significant decrease of 2.0 ± 3.1 degrees **(**Fig. 5c**)**. There was no significant difference in the ATCA between type I and type II orthoses **(**Fig. 5d**)**.

### Forefoot abduction

**Fig. 6:**
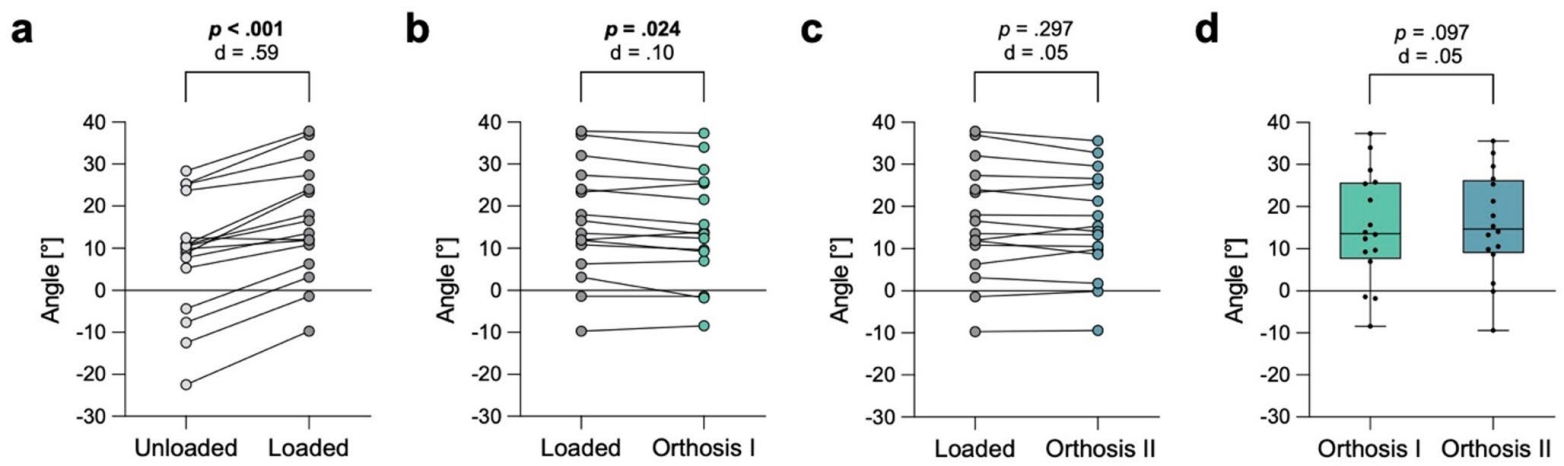
Assessment of forefoot abduction by measuring the axial Meary‘s angle (AMA). **a** Significant increase of the AMA when loading the feet with full body weight. **b** Significant decrease of the AMA when using orthosis type (I) **c** Not significant decrease of the AMA when using orthosis type (II) **d** No significant difference between orthoses type I and II in the AMA. Exact p-values with corresponding Cohen’s d effect size are displayed

Loading the feet without orthoses resulted in a significant increase of the AMA by 8.2 ± 4.3 degrees (Fig. 6a). The use of the type I orthosis led to a significant decrease of the AMA by 1.3 ± 2.0 degrees (Fig. 6b). The use of type II orthosis resulted in a non-significant decrease of 0.6 ± 2.3 degrees (Fig. 6c). However, there was no significant difference in the AMA between type I and II orthoses (Fig. 6d). A presentation of the specific values for the tested angles is also shown in Table 1.

**Table 1:** Absolute values of the respective angles without and with the patient’s full weight and with type I and type II orthoses. Mean values in degrees

Parameter	Unloaded	Loaded	Type I orthoses	Type II orthoses
Sagittal Meary’s angle	−8.2	−16.2	−14.2	−13.5
Axial talocalcaneal angle	28.0	30.9	29.1	28.8
Axial Meary’s angle	8.3	16.4	15.2	15.8

## Discussion

Due to the considerable socio-economic and patient-specific costs, and the inconclusive level of evidence for orthotic treatment, this study aimed to evaluate the effect of foot orthoses on PCFD-related parameters applying WBCT as a relatively new and precise technology. In the applied cadaveric model, our aim was to determine whether foot orthoses with and without a metatarsal pad differentially influence foot alignment, in order to better understand their corrective potential and move beyond solely subjective clinical outcomes. Using a custom-made testing machine, we established a method to dynamically analyze PCFD-related parameters in the cadaveric model with high accuracy using WBCT [18, 19, 22].

Using foot orthoses with or without a metatarsal pad had a mean corrective effect of 1.3 to 2.6 degrees on all three alignment parameters assessed, namely collapse of the longitudinal arch, hindfoot alignment, and forefoot abduction. The strongest effect was observed with 2.6 degrees for the longitudinal arch and the weakest effect with 1.3 degrees regarding forefoot abduction. The hindfoot alignment was corrected by a maximum mean of 2.0 degrees. Only orthoses without a metatarsal pad had a significant effect on forefoot abduction; orthoses including a metatarsal pad also corrected forefoot abduction by an average of 0.6 degrees, but the difference was not significant. Since the metatarsal pad was the only difference between insole types I and II, which were otherwise identical in construction, it is most likely that the small magnitude of the effect is a statistical cause rather than a mechanical cause.

In the past, some studies have attempted to test the effect of foot orthoses on various parameters of foot alignment in the cadaveric model. Using magnetic or optoelectronic tracking systems [34–36] or a goniometer [33], the arch configuration was evaluated in different ways with different types of orthoses. In these studies, a significant, albeit rather subtle influence of different orthoses on various parameters of foot configuration was observed, but an exact assessment using tomographic imaging has not yet been applied to this question. Kitaoka et al. described an improvement in arch alignment of < 2%, whereby the hindfoot alignment was not influenced [34]. In addition to an improved arch alignment, Imhauser et al. observed a slight improvement in hindfoot alignment parameters [35]. Tochigi et al. described an improvement in maximum ankle internal rotation by 1 degree, whereby subtalar rotation was not significantly changed [33].

Overall, based on the results described and in review of the literature, it becomes evident that foot orthoses for flexible deformities are suitable for improving the foot alignment, albeit to a small extent. Nevertheless, an improvement in clinical parameters through the use of foot orthoses, such as improved pain and physical performance [9, 10, 37] or improved functional scorings [8], was described before. Consequently, the question arises as to whether these subtle changes are clinically significant and ultimately how foot orthoses develop their effect. It is conceivable, for example, that not so much the alignment correction itself, but rather the bedding and cushioning provided by the orthosis leads to the described subjective improvement in clinical studies. This might lead to relevant future changes regarding the type of orthotic care for patients with PCFD. The possible influence and direct comparison of varying degrees of alignment correction or bedding and cushioning in a clinical context therefore remains an interesting question for future studies.

Regarding the effect of the metatarsal pad, no significant difference between orthoses with and without a metatarsal pad were found in any of the measurements. The additional use of a metatarsal pad thus showed no stronger or weaker correction potential in relation to the typical parameters of PCFD. However, a possible correction of the forefoot alignment with a potential impact on other foot disorders, e.g. the broad field of metatarsalgia, remains a potential question for future studies.

### Limitations

Despite the strengths of this study, certain limitations need to be discussed. PCFD is a clinical condition that can occur at any age and often affects the younger patients. Due to the nature of body donation, the study population was mainly of advanced age and may have exhibited comparatively rigid foot mechanics. Although all the specimens included expressed PCFD-related parameters under load, this may have resulted in limited foot motion, the effects of which may have been more pronounced in a younger study population. This study was conducted on cadavers to apply the relatively novel and precise WBCT technique, as repeated ionizing radiation cannot be applied to living humans solely for research purposes. No simulation of muscle activity was performed, as the authors assume that no model exists that can adequately simulate the complex interaction of the muscles of the lower leg and intrinsic foot muscles in the cadaveric model. Thus, the assessment primarily focused on the passive bone and ligament PCFD components, whereas the active muscular component remains subject to clinical studies. The selected sample size, although relatively small, aligns with common sample sizes used in biomechanical studies involving cadavers [19, 22, 30–33].

## Conclusion

We have demonstrated that foot orthoses with medial support and lateral counter-support, with or without a metatarsal pad, improved all tested PCFD-components of bone alignment in a cadaveric model, although the effect appeared to be small. Our findings open the door for further clinical research deepening the topic. For example, it remains uncertain whether these subtle changes are responsible for improvements in clinical parameters, or if foot orthoses that primarily provide bedding and cushioning—without aiming to correct alignment—might achieve similar outcomes in the clinical setting. The potential influence and direct comparison of different degrees of alignment correction, bedding, and cushioning in a clinical context remain important questions for future studies and may ultimately inform changes in orthotic care for patients with PCFD.

## Data Availability

Data is provided within the manuscript. Further data are available from the corresponding author on reasonable request.
